# Decoding Sepsis-Induced Disseminated Intravascular Coagulation: A Comprehensive Review of Existing and Emerging Therapies

**DOI:** 10.3390/jcm12196128

**Published:** 2023-09-22

**Authors:** Ahsanullah Unar, Lorenzo Bertolino, Fabian Patauner, Raffaella Gallo, Emanuele Durante-Mangoni

**Affiliations:** 1 Department of Precision Medicine, University of Campania ‘L. Vanvitelli’, 80138 Naples, Italy; ahsanullah.ahsanullah@unicampania.it (A.U.); lorenzo.bertolino@unicampania.it (L.B.); fabianpatauner@gmail.com (F.P.); raffaella.galloo@gmail.com (R.G.); 2 Unit of Infectious and Transplant Medicine, AORN Ospedali dei Colli-Monaldi Hospital, 80131 Naples, Italy

**Keywords:** sepsis, disseminated intravascular coagulation, therapy, corticosteroids, recombinant thrombomodulin, vitamin C, platelet transfusion, immunomodulatory therapy, coagulation, thrombosis, anticoagulant therapy

## Abstract

Disseminated intravascular coagulation (DIC) is a recurrent complication of sepsis. Since DIC not only promotes organ dysfunction but also represents a strong prognostic factor, it is important to diagnose DIC as early as possible. When coagulation is activated, fibrinolysis is inhibited, blood thinners are consumed, and a condition is created that promotes blood clotting, making it more difficult for the body to remove fibrin or prevent it from being deposited in the blood vessels. This leads to microvascular thrombosis, which plays a role in organ dysfunction. Despite efforts to understand the underlying mechanisms of sepsis-induced DIC, healthcare providers worldwide still face challenges in effectively treating this condition. In this review, we provide an in-depth analysis of the available strategies for sepsis-induced DIC, considering their effectiveness, limitations, and potential for future advances. Corticosteroids (CS), recombinant thrombomodulin (rTM), vitamin C, fibrinolytic therapy, and platelet transfusion are among the treatments discussed in the review. In addition, we are specifically addressing immunomodulatory therapy (IMT) by investigating treatments such as granulocyte colony-stimulating factor (G-CSF), granulocyte-macrophage colony-stimulating factor (GM-CSF), interferon gamma (IFN-γ), and mesenchymal stem cell therapy (MSC). Finally, we also examined how these therapies might affect COVID-19 cases, which often present with sepsis-induced DIC. The review suggests that targeted experiments with randomization are needed to verify the effectiveness of these treatments and to discover novel approaches to treat sepsis-induced DIC. By increasing our knowledge of sepsis-induced DIC, we can develop targeted treatments that have the potential to save lives and improve outcomes.

## 1. Introduction

**Figure 1:**
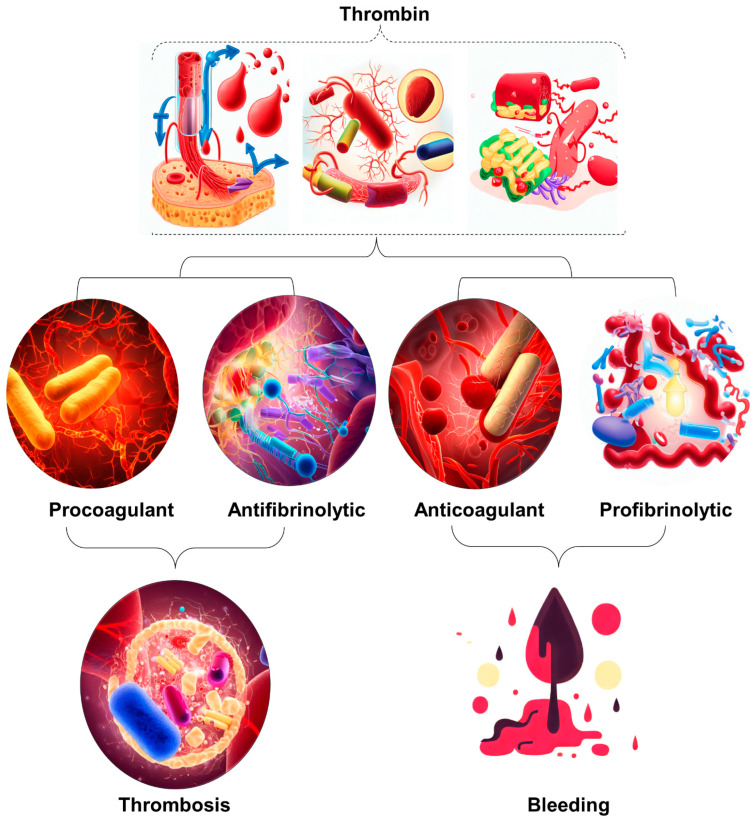
Illustration of the occurrence of excessive thrombin formation in DIC resulting in either bleeding or thrombosis. The specific outcome is determined by the predominant change disrupting the delicate balance between procoagulant and fibrinolytic effects. The dynamic interaction between procoagulant and fibrinolytic mechanisms in DIC plays a crucial role in determining the clinical manifestations of the disease. Therefore, it is imperative to implement timely and targeted therapeutic strategies to maximize patient outcomes.

Disseminated intravascular coagulation (DIC) is a pathological disease that often manifests as a complication in patients with sepsis. Sepsis is a systemic inflammatory response caused by infection and is a major public health concern worldwide [1]. To understand the evolution of the sepsis concept, Table 1 provides an overview of the differences between the traditional approach based on systemic inflammatory response syndrome (SIRS) and the sepsis-3 definition, which emphasizes organ dysfunction or risk of death [1,2,3,4,5,6]. Coagulation disorders that can lead to the development of DIC are often observed in sepsis. DIC is a disease that results in microvascular coagulation, decreased organ perfusion, organ failure, and an increased risk of death. The incidence rate of DIC is estimated at 2.5 cases per 1000 people, with an 8.7% increase over the two decades [1,3]. Sepsis disrupts the blood coagulation process and leads to disruption of hemostasis; however, among these, DIC represents the most serious complication. Approximately 50–70% of patients suffer from DIC. In approximately 35% of cases, it manifests itself overtly. The diagnosis of DIC typically involves the assessment of coagulation markers but lacks sufficient specificity. Therefore, it is crucial to distinguish DIC from diseases characterized by platelet count [7,8]. Unfortunately, several patients who develop thrombocytopenia from a variety of causes are often initially misdiagnosed as having disseminated DIC. This misdiagnosis can result in these patients not receiving the treatment they need. The coagulation process is closely intertwined with the system and is linked to other inflammatory responses [9,10]. The term immune thrombosis refers to the interaction between coagulation and innate immunity [11]. Traditionally, it has been assumed that coagulation activation is triggered by a tissue factor on monocytes and macrophages that is induced by microorganisms and their components, so-called pathogen-associated molecular patterns (PAMPs) [12].Tissue factor (TF) is a potent initiator of coagulation [13] and induces proinflammatory responses through the activation of protease-activated receptors (PARs) [13,14]. Phosphatidylserine on the cell membrane has been identified as an important coagulation activator [15]. Apart from these PAMPs, it has also been found that damage-associated molecular patterns (DAMPs) released by injured cells, such as B. cell-free DNA histones and high mobility group box one protein (HMGB1), contribute to the initiation of coagulation [9]. Extracellular neutrophil traps (NETs), composed of DNA fibers, nuclear proteins, and antimicrobial peptides, have been found to enhance thrombogenicity [9].In addition to activation of coagulation, suppression of fibrinolysis is an important feature of sepsis DIC. PAI-1 released from damaged endothelial cells inhibits fibrinolysis and leads to the development of a thrombotic phenotype associated with coagulopathy (Figure 1) [16,17]. 

This paper explains the need for efficient treatments for sepsis-induced DIC and presents different theories on the mechanisms linking sepsis and DIC. It examines various therapies, such as corticosteroids, recombinant thrombomodulin, vitamin C, fibrinolytic therapy, platelet transfusion, and immunomodulatory therapies, and examines their efficacy, limitations, and potential for future advances. The review also examines the impact of these therapies on severe COVID-19 cases. The paper underscores the importance of high-quality, randomized controlled trials to validate these therapies and demonstrates the potential for developing new therapeutic targets. Overall, the review aims to guide the development of more targeted and effective treatments to reduce the global health burden of sepsis and DIC.

## 2. Comparative Analysis of DIC Diagnosis and Treatment: Eastern vs. Western Approaches

**Figure 2:**
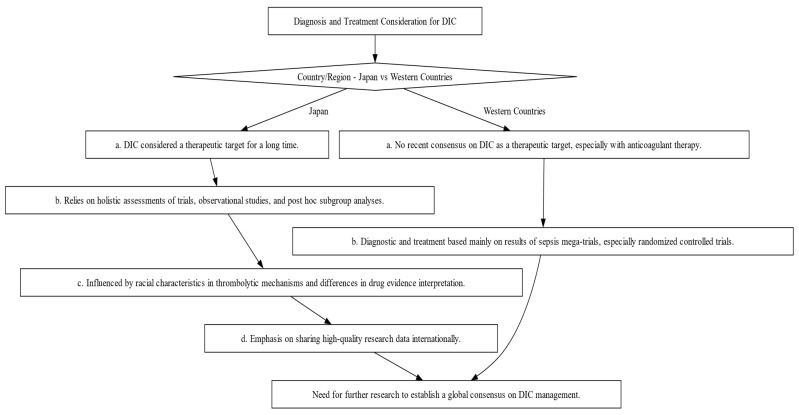
Decision-making Flowchart Depicting the Contrasts in Diagnosis and Treatment Approaches for DIC between Japan and Western Countries. This flowchart illustrates the divergent philosophies and methods for DIC diagnosis and treatment, emphasizing the influence of regional factors such as evidence interpretation and trial designs.

**Figure 3:**
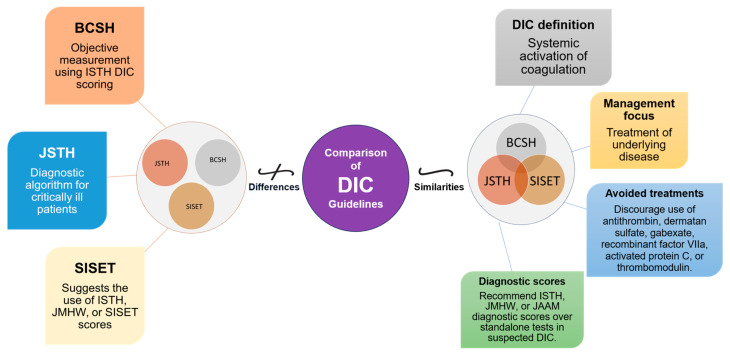
Comparative Overview of DIC Guidelines: Commonalities and Distinctions. This figure illustrates the commonalities and distinctions between DIC guidelines from BCSH (British Committee for Standards in Haematology), JSTH (Japanese Society of Thrombosis and Hemostasis), and SISET (Italian Society for Thrombosis and Haemostasis). Shared principles encompass recognizing DIC as a systemic coagulation activation syndrome with microvascular thrombosis and organ dysfunction, prioritizing treatment of the underlying trigger, and discouraging specific interventions. In suspected DIC cases, all guidelines favor established diagnostic scores (International Society on Thrombosis and Haemostasis (ISTH), the Japanese Ministry of Health and Welfare (JMHW), and the Japanese Association for Acute Medicine (JAAM)). Differences include variations in treatment recommendations, the ISTH’s simple scoring system for overt DIC, JAAM’s focus on critically ill patients, SISET’s endorsement of diagnostic scores, and BCSH’s objective measurement using ISTH DIC scoring system, which is closely linked to clinical outcomes.

The diagnosis and management of DIC manifest distinct variations between Japan and Western countries (Figure 2). These variations are shaped by multiple factors, including differing understandings of thrombolytic mechanisms and the types of evidence deemed valid for therapeutic decision-making. In Japan, clinicians adopt a holistic approach, integrating a wide array of research methodologies, ranging from clinical trials and subgroup analyses to observational studies, to inform treatment protocols [19,20]. Conversely, Western medical practice primarily relies on large-scale studies that focus on sepsis, often employing randomized controlled trials (RCTs) as the research design [19]. In this section, we shed light on these distinctions and their implications and as well as highlight the primary commonalities and distinctions in the clinical guidelines for managing DIC as laid out by BCSH (British Committee for Standards in Haematology), JSTH (Japanese Society of Thrombosis and Hemostasis), and SISET (Italian Society for Thrombosis and Hemostasis (Figure 3) [19,20,21]. The International Society on Thrombosis and Haemostasis (ISTH) has established specific criteria for the diagnosis of overt DIC, which include parameters such as low platelet count and prolonged prothrombin time. In contrast, Japan introduced an alternative approach in 2006 called the Japanese Society of Acute Medicine (JAAM) criteria, which emphasizes laboratory tests and clinical data for an accurate diagnosis.

A comparative study by Gando et al. found that the JAAM criteria have higher sensitivity compared to the ISTH criteria. Sensitivity here means that JAAM criteria are better able to correctly identify DIC cases. In their study, the JAAM criteria diagnosed DIC in 46.8% of cases, while the ISTH criteria identified it in only 18.1%. It is important that all cases identified according to ISTH criteria were also recorded according to JAAM criteria. When looking at 28-day mortality rates, both criteria showed similar results, with 31.8% for JAAM and 30.1% for ISTH [22,23]. This suggests that patients excluded by ISTH criteria may suffer from DIC, highlighting the value of JAAM criteria due to their integrative approach. However, the landscape changed with the introduction of the Sepsis-3 definition, which includes the Systemic Inflammatory Response Syndrome (SIRS) score, making the JAAM criteria somewhat less relevant. In response, a new set of criteria called sepsis-induced coagulopathy (SIC) was developed in 2017 to support early DIC diagnosis in sepsis patients. It considers both sepsis and clotting problems, such as a low platelet count. In diagnosing and managing DIC, physicians rely on laboratory findings, including low platelet count, elevated D-dimers, and abnormal clotting times, alongside clinical assessment [24,25]. These indicators inform the ISTH scoring system for overt DIC diagnosis [2,3]. Key tests include Complete Blood Count (CBC), Partial Thromboplastin Time (PTT), Prothrombin Time (PT) assay, fibrinogen, and D-dimer assays. D-dimer and Fibrin Degradation Product (FDP) tests offer robust diagnostic value [4]. A comprehensive DIC panel includes D-dimer and FDP for swift diagnosis and antithrombin for severity assessment and prognosis [24,25,26]. Table 2 provides a detailed comparison of the diagnostic criteria used by the ISTH for both open DIC and SIC and the criteria used by the JAAM for DIC. The criteria are divided into low-risk, medium-risk, and high-risk categories, each of which has a specific rating [21,22,23,27,28,29,30].

Managing DIC is a multifaceted challenge, and the primary goal is to address the underlying infection. However, there’s limited solid evidence supporting the use of anticoagulant therapy alongside antibiotics and source control. Several large-scale randomized controlled trials (RCTs) involving different anticoagulants have failed to provide conclusive evidence of their effectiveness. Nonetheless, guidelines recommend anticoagulant therapy for patients with severe sepsis-associated DIC, particularly if they have coagulation problems. These recommendations have been validated by the ISTH DIC Scientific Standardization Committee [1,21,27,31]. A hypothesis known as the “East Asian paradox” suggests that unique genetic or environmental factors specific to East Asian populations might contribute to the observed differences in DIC diagnosis and management. The choice of anticoagulant therapy for sepsis-induced DIC depends on the condition’s severity and the specific diagnostic criteria in use. Japanese researchers are encouraged to share their clinical research data internationally to improve our understanding of DIC and its management [30]. Overall, there is a major discrepancy between diagnostic and treatment options in Japan and Western countries, and further research is needed to establish an international consensus on DIC as a therapeutic target. Investigations should also focus on the coagulofibrinolytic system in sepsis and the impact of racial characteristics on thrombolytic mechanisms.

## 3. Can Sepsis-Induced DIC Patients Benefit from Corticosteroids?

Corticosteroids, commonly referred to as CS, continue to be a subject of debate within the community regarding their use in treating sepsis, septic shock, and DIC. There is no consensus due to conflicting results from studies. Gibbison et al. [32] Annane et al. [33] Salluh et al. [34] share perceptions about the possible advantages of CS in addressing sepsis-induced DIC. However, they also highlight the need for research considering the nature of the current evidence. There is obviously a need for more high-quality randomized controlled trials based on these two comprehensive reviews, reflecting the existing uncertainty in this area. In contrast, Rochwerg et al. [35] reported that sepsis patients treated with CS may have reduced mortality. However, they also noted the low reliability of these results, consistent with the urge for more detailed investigation expressed in earlier studies. In addition, according to Gibbison et al. [32], there is quite a positive response to CS on coagulation factors. Despite the possibility of a positive result, the researchers emphasized the experimental nature of their findings and the need for future studies to confirm them. A positive perspective was provided by Gazzaniga et al. [36], who suggested a potential reduction in the risk of SIC with the use of CS. However, like the other studies, they acknowledged the moderate quality of their evidence, further underscoring the need for higher-quality studies. Finally, Ni et al. [37] and Liang et al. [38] found potential benefits of CS in reducing mortality in patients with septic shock and improving outcomes in patients with sepsis-induced coagulopathy. Despite these encouraging results, both studies recommended further investigation and careful interpretation of the results due to possible confounding factors.

Considering these diverse studies, it becomes clear that while there are hints of potential benefits associated with CS in sepsis and septic shock treatment, the current evidence is still inconclusive, with questions about the certainty and quality of the findings. The consensus among all authors is the pressing need for further rigorous and high-quality research to substantiate these preliminary findings, assess the potential risks and benefits more robustly, and clarify the role of CS in the treatment of sepsis and sepsis-induced coagulopathy.

Moreover, future research should consider the factors that might explain the discrepancies in these studies’ results, such as variations in dosage, timing of administration, patient population, patient endotypes, and study design (Table 3). Until a clear consensus appears from more definitive research, clinicians should make decisions on the use of CS on a case-by-case basis, considering each patient’s individual circumstances, the potential benefits and risks of corticosteroid use, and the existing guidelines.

## 4. Evidence from Studies: Rtm’s Effectiveness in Sepsis-Induced DIC

In the context of sepsis-induced DIC, thrombomodulin alfa (rTM), a recombinant form of human thrombomodulin, has been investigated as a potential therapeutic intervention. The mechanism of action of rTM involves its binding to thrombin, leading to the activation of protein C, an anticoagulant that plays a role in the downregulation of the coagulation cascade [8]. Assorted studies were conducted to investigate the efficacy of rTM in the treatment of DIC produced by sepsis. Valeriani et al. [52] conducted a meta-analysis that included data from randomized controlled trials. The research showed a notable decrease in overall mortality among patients who received treatment with rTM compared to the control group [52]. The results of this study indicate that the use of rTM reduced 28-day mortality rates while mitigating bleeding problems. Bleeding complications are often seen as important adverse events in the context of clotting therapy [43,52]. The research performed by Toshiaki Iba and his colleagues provides more evidence to support the effectiveness of rTM as a therapeutic intervention. In the phase III trial, it was shown that although the overall decrease in death rate did not reach statistical significance, a notable decrease in mortality was observed specifically among patients who presented with SIC at the beginning of the study. This implies that rTM may have enhanced efficacy in patients who already demonstrate systemic inflammatory response syndrome (SIRS) at the initiation of therapy [88]. Despite the encouraging results, more assessment is needed for various aspects of rTM use, including the optimal dose, time of application, effectiveness in different patient subgroups, and discussions on cost-efficiency (Table 3). The review of these inquiries will be of paramount importance in fully exploiting the potential of rTM as a therapeutic intervention for sepsis-induced DIC [53].

In general, the existing body of literature, encompassing the studies conducted by Yang et al., Yamakawa et al., and Toshiaki Iba, indicates a potentially advantageous function of rTM in the management of DIC generated by sepsis. Nevertheless, further study is required to enhance the application protocols of rTM, obtain a comprehensive understanding of its long-term effects, and assess its cost-effectiveness. Such efforts will facilitate the translation of these scientific discoveries into advantageous consequences for patients [88].

## 5. Vitamin C in Sepsis and DIC: Promise or Paradox?

The investigation and discussion of vitamin C’s role and benefit in sepsis and DIC treatment has attracted significant medical attention. The putative antioxidant, anti-inflammatory, and anticoagulant properties of vitamin C point to its likely benefits in mitigating organ failure and death rates among people afflicted with these ailments [56,57,58]. However, the existing body of scientific literature presents a multifaceted and contradictory image, emphasizing the need for further research. According to a systematic review and meta-analysis conducted by researchers [59], the administration of vitamin C did not provide a statistically significant reduction in the mortality rate, length of stay in the intensive care unit (ICU), or duration of mechanical breathing among patients with sepsis. Nevertheless, the study showed a discernible inclination toward diminishing the length of vasopressor medication, hence implying that vitamin C may influence the disease’s progression. A recent scientific publication [61] highlighted the possible benefits of vitamin C in the treatment of sepsis, especially focusing on its antioxidant properties and ability to regulate the immunological response. Nevertheless, the authors also placed significant emphasis on the equivocal nature of the existing data and advocated for more studies to ascertain the most effective dosage and timing of vitamin C delivery in individuals with sepsis [61]. Research conducted on the use of high-dose vitamin C (HDVC) therapy in the treatment of sepsis and DIC has shown varied outcomes. A comprehensive investigation revealed that the administration of intravenous vitamin C (IVVC) resulted in a notable enhancement of the delta Sequential Organ Failure Assessment (SOFA) score and a decrease in the duration of vasopressor utilization among individuals diagnosed with sepsis or septic shock. However, no significant correlation was observed between the use of IVVC and a reduction in short-term death rates [57]. Nevertheless, the Vitamin C Therapy for Routine Care in Septic Shock (ViCTOR) trial concluded that the intravenous administration of a regimen consisting of vitamin C, thiamine, and hydrocortisone did not lead to a significant decrease in overall mortality in patients afflicted with septic shock. However, it was noted that the time needed to reverse the onset of septic shock was markedly reduced in the patient group that received this combination therapy [60]. These results were consistent with recent randomized controlled trials (RCTs) that also reported no mortality benefit with combination therapy [89,90,91,92,93,94]. Another study revealed an intriguing finding. It showed that adult patients dealing with sepsis and undergoing vasopressor therapy within the ICU, when treated with intravenous vitamin C, faced an increased risk. In contrast to those who were administered a placebo, those who received active treatment had an increased probability of experiencing either fatality or persistent organ failure. The heightened risk endured for a substantial duration, namely, until the 28th day following the initiation of the therapy [54]. The divergent results highlight the intricate nature of the involvement of vitamin C in the treatment of sepsis and DIC. While several studies have shown potential advantages, others have discovered no substantial effect or even possible negative consequences [54,55]. This underscores the need for more rigorous, randomized, controlled studies to clarify the therapeutic efficacy of vitamin C in these specific disorders. It is advisable for clinicians to use prudence when considering administering vitamin C for sepsis treatment and DIC, pending the availability of further conclusive data. These findings are significant for researchers in recognizing the complex nature of vitamin C’s role in sepsis. Consequently, there is a need for studies that not only examine its influence on death rates and organ dysfunction but also delve into its impact on the development of the disease and the quality of life experienced by patients. It is important to make efforts to standardize dosage regimens to improve the effectiveness of treatment (Table 3).

In short, the current corpus of literature provides a nuanced and incongruous portrayal of the involvement of vitamin C in the treatment of sepsis and DIC. The continuous discourse among academics and doctors highlights the divergent findings, hence emphasizing the necessity for more exploration. Based on the available information, healthcare professionals are recommended to exercise prudence when considering the administration of vitamin C in cases of sepsis and DIC. The determination of whether to use this intervention should be based on the specific attributes of each patient and the ongoing accumulation of scientific knowledge in this area. The intricacy of biological systems and the difficulties in converting possible therapeutic pathways into practical therapies are highlighted by this argument, particularly for preclinical readers.

## 6. Fibrinolytic Therapy in Sepsis-Induced DIC: A Potential Game-Changer?

The investigation of fibrinolytic treatment, particularly the use of fibrinolytic drugs such as tissue plasminogen activator (tPA), is a subject of research in the context of sepsis-induced DIC. While the administration of fibrinolytic treatment has a potential hazard of inducing bleeding, it also offers promising advantages by mitigating clot formation and enhancing organ perfusion. Numerous investigations have been conducted to examine the use of fibrinolytic medicines in DIC generated by sepsis. Schouten et al. [66] studied the administration of tPA in a baboon model of DIC induced by sepsis. The authors noted that the administration of tPA resulted in an amplification of fibrinolysis, a reduction in microvascular thrombosis, and an improvement in organ perfusion [66]. The authors [65,67] assessed the use of low-dose tPA in patients who developed DIC due to sepsis. Improvements in coagulation abnormalities were observed because of how to manage low-dose tPA treatment in patients with DIC induced by sepsis, as specified by the study results. The observed enhancements included a reduction in the levels of fibrin degradation products and an augmentation in platelet counts. Significantly, the administration of low-dose tPA did not yield any considerable complications related to bleeding. However, it is imperative to recognize that the application of fibrinolytic therapy in sepsis-induced DIC remains a topic of debate due to the simultaneous risk of hemorrhage. Achieving a delicate equilibrium between the inherent hazards associated with bleeding problems and the prospective advantages necessitates prudent patient selection and diligent monitoring.

In the realm of DIC triggered by sepsis, a thorough examination was undertaken by numerous esteemed researchers. This comprehensive review sought to analyze the existing information pertaining to fibrinolytic treatment [62,63,64,65]. In the realm of scholarly inquiry, a group of diligent researchers undertook a recent systematic analysis to meticulously scrutinize the existing data pertaining to the utilization of fibrinolytic treatment in instances characterized by DIC provoked by sepsis. The findings of the analysis suggest that although fibrinolytic therapy may exhibit improvements in certain laboratory markers linked to coagulation and fibrinolysis, its impact on clinical endpoints, such as mortality, remains inconclusive [62,63,64,65]. The necessity for further study is posited by the authors to ascertain the efficacy of fibrinolytic treatment in augmenting patient outcomes in instances of sepsis-induced DIC. Further investigation is warranted concerning the utilization of fibrinolytic therapy, specifically tPA, in the setting of DIC precipitated by sepsis. Despite the potential for bleeding, the administration of fibrinolytic therapy possesses the capability to attenuate the formation of blood clots and augment the perfusion of vital organs. Favorable effects on fibrinolysis and coagulation problems have been associated with the use of fibrinolytic drugs, as indicated by previous research. At present, the definitive impact of these agents on clinical outcomes remains to be conclusively determined. The optimization of fibrinolytic therapy for sepsis-induced DIC necessitates additional inquiry (Table 3).

## 7. Platelet Transfusion in Sepsis-Induced DIC: Navigating Controversy and Conflicting Evidence

In this study, we aim to fully examine the contentious issue surrounding the use of platelet transfusion in the context of sepsis-induced DIC. Therapeutic decision-making can be obscured by contradictory advice within the extensive range of guidelines. In accordance with certain recommendations, it is advisable to consider the possibility of platelet transfusion for patients who are afflicted with sepsis-induced DIC and manifest severe thrombocytopenia or are currently undergoing active bleeding [71]. However, it is vital to recognize the lack of evidence on the effectiveness of platelet transfusion in this context. The existing body of research relies on expert opinion rather than robust scientific trials. A notable investigation in the same domain of the TOPPS trial was conducted by Estcourt et al. [69]. The study’s objective was to evaluate the impact of platelet transfusion on the mortality rate among critically sick patients with thrombocytopenia. The research findings indicated that the administration of platelet transfusion did not have any statistically significant effects on reducing death rates or enhancing patient outcomes [69]. A further investigation was conducted, wherein attention was directed toward individuals suffering from septic shock and DIC [68]. This study’s main objective was to assess the potential association between platelet transfusion and mortality. In congruence with the TOPPS trial, this study demonstrated a lack of mortality reduction in relation to platelet transfusions [68].

This narrative is confirmed by the study conducted by He et al. [70] and Yatabe et al. [72]. The results of their study stipulate that the administration of platelet transfusion did not lead to a reduction in mortality rates among people diagnosed with sepsis. Conversely, it is plausible that this could reduce the duration of stay in the intensive care unit and the hospital, suggesting the potential for unfavorable outcomes [70,72]. In total, the tests collectively demonstrate the lack of empirical evidence of the effectiveness of platelet transfusion in cases of sepsis-induced DIC. Presenting the innovative outlook, Syed Muzaffar et al. offer a contrasting perspective to the studies [73]. The focal point of their research lies in the examination of the pivotal role played by coagulation disorder in the development of sepsis, thereby illuminating the range of coagulopathy induced by sepsis. A more individualized strategy for patient care can be enabled through the proposed triad of coagulation profiles utilizing the Coagulation Index (CI), as put forth by the authors. The authors also underscored the limitations of conventional coagulation assays (CCAs) and drew attention to the utility of thromboelastography (TEG) in the assessment of coagulation profiles [95,96]. The decision to administer platelet transfusion for sepsis-induced DIC should depend on certain contextual factors, considering the patient’s clinical condition, bleeding tendency, and overall trade-off between the advantages and disadvantages of platelet transfusion. The importance of close monitoring of platelet counts and clinical indications in making this decision cannot be overstated. While some guidelines suggest giving platelet transfusion for severe thrombocytopenia or active bleeding, there is also research available, such as the TOPPS test [69] and the study by Andrew et al. [68], that does not demonstrate a significant reduction in mortality or improvement in outcomes from platelet transfusions in this scenario.

Valuable contributions to the existing knowledge of platelet transfusions and coagulation patterns in individuals with sepsis are made by the research conducted by He et al. [70] and Muzaffar et al. [73]. The significance of their study lies in the emphasis on the necessity of implementing meticulous randomized controlled trials and pursuing additional research endeavors to acquire a more comprehensive comprehension of the intricate involvement of platelet transfusion in sepsis-induced DIC. Efforts in future research should be directed toward the identification of distinct subpopulations of patients who may potentially derive advantages from platelet transfusion. Furthermore, the evaluation of their influence on clinical outcomes, encompassing mortality rates and the incidence of hemorrhagic complications, should be undertaken (Table 3).

The ongoing debate and uncertainty surrounding the role of platelet transfusion in the context of sepsis-induced DIC can be attributed to conflicting research findings and divergent viewpoints among experts. There is an ongoing debate within the medical community regarding the pressing necessity for comprehensive research aimed at enhancing our comprehension and expediting the application of clinical therapies in a streamlined fashion. The administration of platelet transfusion in sepsis-induced DIC is currently the subject of a lively debate. This debate stems from the absence of compelling evidence highlighting a noteworthy decrease in mortality rates or enhancement in clinical outcomes linked to this intervention [68,69,70]. Within the realm of clinical decision-making, the utmost significance lies in the prioritization of a customized approach, which is contingent upon meticulous monitoring and diligent evaluation of the unique clinical trajectory exhibited by each patient. To formulate personalized recommendations that align with the specific circumstances of each patient, physicians are required to meticulously assess and modify the intricate equilibrium between potential hazards and expected advantages associated with a transfusion. Valuable insight into the complex elements of the coagulopathy associated with sepsis has been provided by recent developments in scientific studies. The findings of this study lend support to the concept that a uniform treatment strategy may not be the most advantageous but rather emphasize the importance of customizing medical interventions to suit the unique characteristics of each patient (Table 3). In this context, the significance of TEG becomes apparent, as exemplified by its noteworthy application as a diagnostic instrument [73]. Considering the existing dearth of definitive and all-encompassing data, it is important for medical practitioners to exercise judiciousness in contemplating the utilization of platelet transfusion for managing sepsis-induced DIC.

## 8. Immunomodulatory Therapy: G-CSF, GM-CSF, IFN-γ, MSCs in Sepsis, DIC, and Their Implications for Clinical Practice & Severe COVID-19

IMT in the context of sepsis-induced DIC is currently an area of active research [74,88]. Various agents, including granulocyte colony-stimulating factor (G-CSF), granulocyte-macrophage colony-stimulating factor (GM-CSF), and interferon-gamma (IFN-γ), have been investigated for their potential role in sepsis-induced DIC [97,98]. Nevertheless, the precise elucidation of their specific influence on sepsis-induced DIC remains to be definitively established [97,98]. Granulocyte colony-stimulating factor (G-CSF), a hematological growth factor, stimulates the production and mobilization of neutrophils. Conversely, it is worth noting that granulocyte-macrophage colony-stimulating factor (GM-CSF) exerts its influence on both neutrophils and monocytes/macrophages [99]. The drugs under consideration have been the focal point of scientific inquiry, with the objective of examining their capacity to enhance immune functionality and enhance outcomes in instances of DIC precipitated by sepsis [74,75]. The impact of G-CSF treatment on individuals diagnosed with sepsis and DIC was examined by the authors [76,77]. The study’s findings elucidated that the administration of G-CSF led to a noteworthy increase in neutrophil counts and amelioration of coagulation parameters in individuals afflicted with sepsis-induced DIC [76,77]. In the therapeutic management of DIC induced by sepsis, IFN-γ, an immunomodulatory drug, has exhibited promising potential. The impact of IFN-γ treatment on patients with sepsis-induced DIC was examined by Iba T. et al. [53] in their study. The study’s findings revealed that the administration of IFN-γ yielded enhancements in coagulation abnormalities, a decrease in the duration of DIC, and a potential decline in fatality rates [53]. However, further inquiry is imperative to attain a holistic understanding of the role played by immunomodulatory drugs in the context of DIC induced by sepsis. Included in this analysis are the exploration of the mechanisms of action, the determination of the best dosage, the identification of the ideal timing for administration, and the refinement of the criteria for selecting patients who may potentially derive therapeutic benefits from such treatment. Conducting randomized controlled studies [6,8,53,72,99,100,101,102,103] is imperative for assessing the effectiveness, safety, and clinical outcomes associated with the utilization of immunomodulatory drugs in this specific context.

Significant interest has been generated in the treatment of sepsis, transplant medicine, and autoimmune diseases through the utilization of mesenchymal stem cell (MSC) therapy. Although the precise cellular and molecular mechanisms underlying MSC-mediated immunomodulation have not yet been fully elucidated, preclinical studies have demonstrated promising results. The immunomodulatory effects of MSCs are exerted via the secretion of cytokines, a process that can be influenced by both the local microenvironment and inflammatory cytokines [78,79].

Additionally, even apoptotic, metabolically inactivated, or fragmented MSCs possess immunomodulatory potential [79,104]. However, the lack of standardization in the isolation, culture, and characterization of MSCs complicates data comparison. MSCs can be derived from various adult and neonatal tissues, each exhibiting unique features in vitro and in vivo [79,80,81] Notably, freshly thawed MSCs appear to have reduced immunomodulatory capacity compared to continuously cultured MSCs [105]. The local microenvironment plays a critical role in shaping MSC-mediated immunomodulation, further adding to its complexity [82,83]. MSCs exert their immunomodulatory effects through a combination of cell contact-dependent mechanisms and the release of soluble factors [79,83,84,85,106,107,108,109,110,111]. MSCs have a significant impact on various immune cells, particularly anti-inflammatory monocytes/macrophages and regulatory T cells (Tregs) [84,85,86]. Interestingly, MSC viability does not appear to be a prerequisite for some of their immunomodulatory effects, as apoptotic MSCs have demonstrated beneficial effects in animal models [112]. Exploring the use of dead or fragmented MSCs may provide more predictable immunomodulatory effects and facilitate better comparison across studies [112].

A case report by Galic et al. presented the successful management of a 14-month-old [93]. The treatment included a combination of antibiotics, plasmapheresis, dialysis, methylprednisolone, mycophenolate mofetil, and eculizumab [87]. Eculizumab therapy was considered in rare cases of sepsis with massive complement consumption after resolving life-threatening multiorgan failure [87]. These studies and reports have important implications for future clinical practice. IMT, including the use of G-CSF, GM-CSF, IFN-γ, and MSCs, shows promise in improving outcomes in sepsis-induced DIC. However, further research is needed to determine the optimal use, dosing, timing, and impact on clinical outcomes of these therapies [8,53,72,75,78,80,81,82,98,99,100,101,103,104,107,111,113,114,115,116,117,118,119,120,121,122,123,124,125]. The need for cautious administration and withdrawal of the drug is underscored by the potential utilization of eculizumab in instances of sepsis accompanied by complement consumption. In addition, the investigation of extracellular vesicles derived from mesenchymal stem cells (MSC-EVs) as a therapy without the need for cell transplantation presents a hopeful alternative to treatments based on MSCs [87].

By elucidating the intricate relationship between severe COVID-19 and sepsis, which arises from viral infection, one can glean profound insights into the nature of the disease and subsequently enhance the trajectory of future investigations pertaining to therapeutic interventions and preventive strategies [126]. Consideration should be given to the inclusion of IMT in the treatment regimen for severe cases of COVID-19 in conjunction with etiological and supportive interventions [126,127]. The primary objective in the context of COVID-19 immunotherapy should revolve around the mitigation of exaggerated inflammatory responses while simultaneously preserving a controlled level of inflammation and facilitating the restoration of profound immunosuppression [126,127]. In severe cases of COVID-19, it is imperative to conduct additional research to substantiate the safety, efficacy, timing, and dosing of IMT (Table 3) [126]. 

In conclusion, IMT and MSC-based treatments hold promise for improving outcomes in sepsis-induced DIC and other conditions. However, further research is necessary to fully understand their mechanisms of action, optimize their use in clinical practice, and ensure their safety and efficacy. The exploration of MSC-EVs as a cell-free therapy and the consideration of sepsis-like features in severe COVID-19 provide valuable insights for future studies and therapeutic developments.

## 9. The Controversy Surrounding Anticoagulant Therapy for Sepsis and DIC Management: An In-Depth Analysis 

Blood clotting problems are common in sepsis and DIC, which can lead to organ failure and death. The effectiveness of using anticoagulant therapy to treat SIC and DIC remains a controversial topic among researchers [27,128]. A comprehensive analysis by Freeman et al. found no clear survival benefits from anticoagulant therapy in the general sepsis population and even suggested a higher risk of bleeding complications [129]. Some studies examined the concomitant use of low-dose heparin for the prevention of deep vein thrombosis and evaluated various outcomes, including all-cause mortality, at different time points [130].

### 9.1. Summary of Previous Research

**Figure 4:**
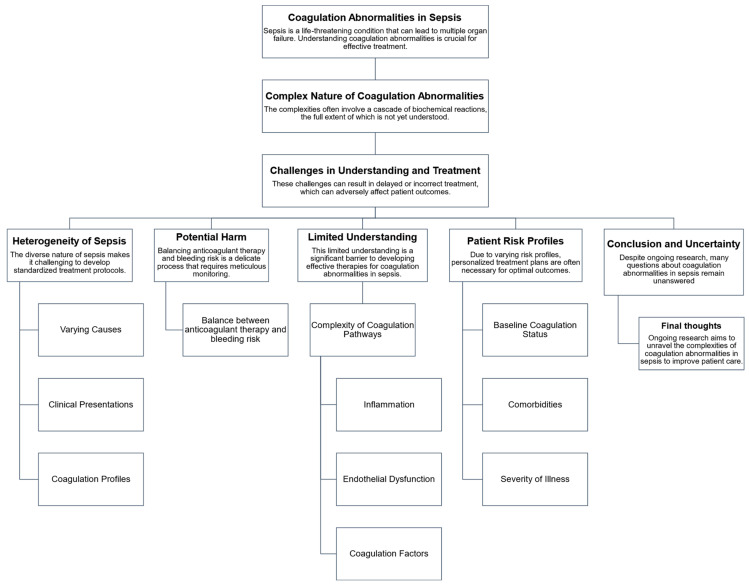
Understanding coagulation disorders in sepsis. This flowchart provides a structured overview of the complexities and challenges associated with understanding and treating coagulation disorders in sepsis. It describes the complexity of coagulation disorders, the challenges of diagnosis and treatment, and the factors that complicate medical intervention in this area. The graphic also highlights the need for personalized treatment plans due to different patient risk profiles and concludes with a note on ongoing research aimed at unraveling these complexities.

This section reviews previous studies to provide a comprehensive overview of the effectiveness of anticoagulant therapy in sepsis treatment (Figure 4). When reviewing [27,53,131] various studies [6,8,132,133,134], it becomes clear that the results derived from these studies were based predominantly on short-term observations. It is important to recognize that these short-term results may not necessarily reflect the long-term dynamics of sepsis in humans. Therefore, when considering the use of anticoagulant therapy, it is essential to strike a balance between the potential benefits and associated side effects. Furthermore, the authors of these studies have wisely identified certain critical gaps in the existing research landscape. A notable omission concerns the lack of consideration of the cost-effectiveness of anticoagulant therapies, particularly in settings outside Japan (Table 4) [131]. This is very worrying as the economic impact of such therapies is often overlooked. Furthermore, the authors highlighted several issues that contribute to the heterogeneity observed in different studies. These issues include issues such as the risk of bias, the criteria for defining bleeding complications, and differences in the types of anticoagulants used in individual research efforts [27,128,129,131,135,136]. It is important to understand these factors because they can have a significant impact on the interpretation of study results. A review of previous research highlights the need for a more nuanced and comprehensive approach when considering anticoagulant therapy for sepsis treatment [131,137,138]. The aim is to examine not only the immediate effects but also to consider the possible long-term effects, cost-effectiveness, and factors that contribute to the heterogeneity of the studies. Such considerations are critical to advancing our understanding of the utility of anticoagulant therapy in the context of sepsis treatment.

### 9.2. Study Hypothesis, Significance, and Unanswered Questions

In this section, we address the hypothesis, significance, and open questions surrounding the use of anticoagulant therapy in sepsis treatment (Table 3 and Table 4 and Figure 2). The present study examines the potential benefits of anticoagulant therapy in patients with SIC or DIC, with a focus on specific subgroups of individuals. However, it is important to recognize that despite some studies indicating favorable outcomes, the totality of the evidence does not provide clear support for the universal use of anticoagulant treatment in the broader sepsis population [27,30,137]. Therefore, the question of whether anticoagulant therapy is generally beneficial for sepsis patients remains a controversial topic in the scientific community. Anticoagulation plays a central role in the body’s innate defense mechanisms against infections [139,140]. However, widespread, and indiscriminate use is discouraged due to the increased risk of bleeding. In addition, there are concerns about possible impairment of pathogen clearance due to excessive coagulation [139]. A constant challenge in this area is, therefore, the establishment of precise criteria for patient selection. This challenge is critical to ensure that anticoagulation therapy is administered to those who can derive the greatest benefit from it. The debate surrounding the use of anticoagulation therapy in sepsis patients highlights the complexity of this medical intervention. As researchers continue to study and elucidate the potential benefits, the need for a differentiated approach to patient selection and treatment remains a primary focus of future research efforts [141,142,143].

### 9.3. Antithrombin and Combination Therapy

This section focuses on the role of antithrombin, an important endogenous anticoagulant, and explores the potential of combination therapy (Table 4 and Table 5) [144]. Antithrombin has been the subject of extensive investigation in the context of sepsis-induced coagulopathy. However, it is important to emphasize that relying on a single study alone to make a final decision about effectiveness is not conclusive. A notable phase 4 study conducted in patients with septic DIC provided results worth considering. This study found no improvement in 28-day mortality associated with the use of antithrombin in this population [128,131,137,140,141,142,143,145,146,147]. Interestingly, there is growing interest in the concept of combination therapy. This approach is promising because both the antithrombin GAG system and the thrombomodulin protein C system exhibit significant downregulation and function independently in sepsis-induced coagulopathy [146,148,149]. It is important to note that while clinical trials have not yet been completed in this specific context, preliminary investigations into combining these therapeutic approaches have shown potential. These early studies suggest that combination therapy, compared to individual therapeutic interventions, may be key to ameliorating organ damage and ultimately improving survival rates [130]. These results highlight the evolving landscape of sepsis treatment and highlight the need for further research to refine therapeutic strategies and improve patient outcomes in severe sepsis and coagulation disorders.

### 9.4. Optimal Therapeutic Targets and Considerations in Anticoagulant Therapy

In the domain of anticoagulant therapy, the choice of therapeutic targets is contingent upon clinical variables and the specific anticoagulant employed. Notably, patients with mechanical heart valves necessitate a targeted International Normalized Ratio (INR) range between 2.5 and 4.9, with a recommended target INR of 3.0 to 4.0 [153], whereas those undergoing vitamin K antagonist therapy are advised to achieve a target INR of 2.0 to 3.0 [154]. However, the optimal therapeutic targets for antithrombin (AT) and recombinant thrombomodulin (rTM) therapies remain less well-defined. Furthermore, the utilization of target-specific oral anticoagulants (TSOACs) such as dabigatran, apixaban, and rivaroxaban necessitates meticulous patient assessment to determine their suitability in the clinical context [155,156]. Among the array of anticoagulant options available, unfractionated heparin offers rapid onset and a brief half-life, low-molecular-weight heparin presents a longer half-life and subcutaneous administration [157,158], while fondaparinux is a synthetic pentasaccharide with selective antithrombin binding and factor Xa inhibition. Warfarin, functioning as a vitamin K antagonist, inhibits the synthesis of vitamin K-dependent clotting factors, and direct thrombin inhibitors (e.g., dabigatran, argatroban, desirudin, bivalirudin) directly obstruct thrombin activity [158,159,160]. Factor Xa inhibitors (e.g., rivaroxaban, apixaban, edoxaban, betrixaban) belong to a distinct category, inhibiting factor Xa. The choice of anticoagulant therapy hinges upon the patient’s clinical presentation, the indication for therapy, and the clinical expertise of the healthcare provider, all within the framework of established treatment guidelines. The primary concern associated with anticoagulant therapy is the risk of bleeding, which can manifest in various anatomical locations and pose significant clinical challenges. Additionally, patients may experience a spectrum of potential side effects, including gastrointestinal symptoms, dizziness, headaches, dermatological issues, hair loss, jaundice, and skin hemorrhage. Regular blood tests to monitor clotting time are requisite for patients receiving warfarin to assess bleeding risk. In cases where signs of excessive bleeding, such as hematuria or prolonged nosebleeds, become evident, prompt medical attention is imperative. Consequently, comprehensive discussions between patients and their healthcare providers regarding potential side effects should precede the initiation of anticoagulant therapy to facilitate informed decision-making and optimize patient outcomes [140,161].

### 9.5. Biomarkers, Machine Learning, and Emerging Trends in Sepsis Diagnosis and DIC Management

Recent research [30,140,162,163] focused on identifying biomarkers for sepsis diagnosis to address the complex heterogeneity of sepsis and its diverse clinical presentation, encompassing various clinical signs and symptoms. Biomarkers like C-reactive protein (CRP) [164], procalcitonin (PCT) [165], interleukin-6 (IL-6) [166], CD64 [165,167], presepsin [165], and soluble TREM-1 (sTREM-1) [168] have displayed promise in sepsis diagnosis. However, their practical application is challenged by the multifaceted nature of sepsis. Finding the ideal sepsis-specific biomarker remains a challenge, with multiple biomarkers potentially working in concert to identify critically ill patients in need of closer monitoring. Host response biomarkers play a critical role in diagnosis, early organ dysfunction recognition, risk assessment, prognosis, and patient management, including antibiotic stewardship [168].

Machine learning has emerged as a potent tool for biomarker discovery, enabling the identification of novel diagnostic and prognostic biomarkers by analyzing extensive datasets with intricate relationships [169,170,171]. Machine learning algorithms like Random Forest, Support Vector Machines (SVM), Artificial Neural Networks (ANN), Logistic Regression, and Decision Trees have been employed to sift through vast datasets, revealing patterns and relationships not readily discernible through conventional analysis [163,165,170,172]. Ensuring high-quality and representative training data for machine learning algorithms is imperative [165]. Additionally, the clinical relevance and interpretability of machine learning models are critical considerations, necessitating validation with independent datasets to ensure their applicability in patient care [168]. The choice of a specific machine learning algorithm depends on the research question and dataset characteristics [167,171]. As research in sepsis biomarkers and machine learning progresses, these innovative approaches hold promise for enhancing sepsis diagnosis and patient care.

### 9.6. Challenges and Future Perspectives

This section reviews the complexities, challenges, and potential future directions associated with anticoagulant therapy in the context of septic coagulopathy (Table 5). Anticoagulant therapy, although promising, has significant disadvantages, including high cost and increased risk of bleeding [131]. It is important to recognize that anticoagulant therapy consistently shows a trend toward bleeding events compared to placebo, with bleeding occurring more than 1.7 times more frequently in the treatment group. Unfortunately, these bleeding events often negate the benefit of anticoagulants. In addition, it is important to understand that the risk of bleeding may vary depending on the inhibitors used, their concentration, how long they remain in the body and the associated disorders in the body’s blood clotting system. Therefore, it is critical to carefully select patients who are most likely to benefit from this treatment due to persistent increased bleeding risk. The challenges associated with the use of anticoagulant therapy as a treatment strategy become apparent when examining the problems faced by randomized controlled trials (RCTs) examining its use in sepsis. The treatment of coagulation problems in sepsis patients should be approached with great care and considered in the broader context of individualized patient care. The difficulties observed in RCTs investigating anticoagulation therapy in sepsis highlight the need for a more differentiated and personalized approach. As our understanding of sepsis and its coagulation aspects continues to improve, we can expect to see the development of more refined treatment approaches. These encouraging developments hold the potential to significantly improve the treatment of this complicated and challenging medical condition.

## 10. Conclusions

With limited viable treatment strategies, sepsis-related DIC continues to pose a complex and perilous challenge. The management of this condition remains complex and life-threatening. Clarification of the pathological mechanisms behind sepsis and DIC, coupled with advances in therapeutic interventions, can achieve favorable clinical results in affected individuals. Additional high-quality randomized controlled trials are needed to mitigate the efficacy and controversies surrounding the role of anticoagulant therapy, which encompasses the utilization of heparin, recombinant activated protein C (APC), and antithrombin (AT). Blood purification methods such as polymyxin B hemoperfusion and immunotherapy approaches such as reversing immune function with drugs or antibodies have shown some potential, but further investigation is needed to assess their efficacy and safety. The current state of research on sepsis treatment DIC underscores the need for multidisciplinary approaches and collaborations between clinicians, researchers, and healthcare organizations to improve patient outcomes. Amid the ongoing global pandemic, the integration of these therapies, notoriously IMT, is crucial for treating severe COVID-19 cases presenting as sepsis-induced DIC. Further studies are required to identify new therapeutic targets and improve our understanding of the underlying mechanisms of sepsis DIC. By continually developing our knowledge of sepsis DIC, we can work to expand effective and targeted therapies that can save lives and improve patient outcomes.

## Figures and Tables

**Table 1:** A Comparative Analysis of Sepsis Definitions: Traditional SIRS-based vs. Sepsis 3 Approach [18].

Feature	Previous Sepsis Definitions (SIRS-Based)	Sepsis 3 Definition
Definition	Sepsis is SIRS + confirmed or presumed infections *	Sepsis is life-threatening organ dysfunction due to a dysregulated host response to infection
Organ Dysfunction Criteria	Based on individual clinical criteria (e.g., temperature, heart rate, respiratory rate, WBC count)	Organ dysfunction defined as an increase of 2 or more points in the Sequential Organ Failure Assessment (SOFA) score
Clinical Criteria	Relatively simple criteria (e.g., T > 38 C or <36 C, *p* > 90/min, RR > 20/min or PaCO_2_ < 32 mmHg, WBC > 12 or >10% immature band forms)	qSOFA (HAT) **: Hypotension (SBP ≤ 100 mmHg), Altered mental status (any GCS < 15), Tachypnea (RR ≥ 22)
Classification of Severity	Sepsis, Severe Sepsis, Septic Shock	Sepsis, Septic Shock (Severe Sepsis no longer exists)
Diagnostic Accuracy	Lack of sensitivity and specificity for diagnosing severe sepsis	Improved predictive validity and accuracy in diagnosing sepsis
Use in ICU Patients	SIRS criteria lacked sensitivity for defining sepsis in ICU patients	SOFA score superior to SIRS in predicting mortality in ICU patients
Use in Non-ICU Patients	Less accurate in predicting hospital mortality outside the ICU	Similar predictive performance in non-ICU patients
Global Applicability	Used globally, but lacks standardization and content validity	Development and validation conducted in high-income countries
Prognostic Value	Limited ability to predict patient outcomes and mortality	Enhanced ability to prognosticate patient outcomes and mortality risk
Emphasis on Infection Trigger	Inclusion of infection as a crucial component in sepsis diagnosis	Maintains the importance of infection in defining sepsis
Endorsement by Professional Orgs.	Various organizations endorsed previous definitions	Not universally endorsed by all organizations

T > Temperature, *p* > Pulse Rate, RR > Respiratory Rate, Pa-CO_2_ > Partial Pressure of Carbon Dioxide (Pa-CO_2_), WBC > White Blood Cell Count. qSOFA > quick Sequential Organ Failure Assessment, “HAT” represents the three components of qSOFA: H-Hypotension, A-Altered Mental Status. T–Tachypnea. * Sepsis is characterized by Systemic Inflammatory Response Syndrome (SIRS) accompanied by confirmed or presumed infections. ** qSOFA is a simplified bedside tool that aids healthcare providers in quickly assessing patients with suspected infection for signs of organ dysfunction. If a patient presents with two or more of the qSOFA criteria, it indicates a higher risk of sepsis-related complications and may prompt further evaluation and early intervention to improve patient outcomes. However, it is important to note that qSOFA is not intended to diagnose sepsis definitively but serves as a screening tool to identify patients who require closer monitoring and additional evaluation for possible sepsis.

**Table 2:** Comparative Evaluation of Diagnostic Criteria Across ISTH Overt DIC, JAAM DIC, and ISTH SIC Scoring Systems.

**Parameter (Units)**	**Diagnostic Method**	**Low-Risk Criteria (Score = 1)**	**Moderate-Risk Criteria (Score = 2)**	**High-Risk Criteria (Score = 3)**	**Interpretative Notes**
Platelet Count (×10⁹ per L)	ISTH Overt DIC	50–100	N/A	<80 or 50% drop in 24 h ^1^	Lower counts indicate severe clotting issues
	JAAM DIC	<50	N/A	N/A	-
	ISTH SIC	100–150	<100	N/A	-
Fibrin Degradation Products (FDP)/D-dimer (μg/mL)	ISTH Overt DIC	N/A	Moderate increase ^2^	Strong increase ^3^	Elevated levels suggest severe clotting issues
	JAAM DIC	10–25	N/A	≥25	-
	ISTH SIC	N/A	N/A	N/A	-
Prothrombin Time (PT) (seconds or PT-INR)	ISTH Overt DIC	1.2–1.4 PT-INR	3–6 s	≥6 s	Longer times signify clotting dysfunction
	JAAM DIC	1.2–1.4 PT-INR	N/A	>1.4 PT-INR	-
	ISTH SIC	N/A	N/A	N/A	-
Fibrinogen Levels (g/mL)	ISTH Overt DIC	N/A	N/A	<100	Low levels indicate severe coagulation issues
	JAAM DIC	N/A	N/A	N/A	-
	ISTH SIC	N/A	N/A	N/A	-
SIRS Score	ISTH Overt DIC	N/A	N/A	N/A	-
	JAAM DIC	>3	N/A	N/A	Elevated scores indicate systemic inflammation
	ISTH SIC	N/A	N/A	N/A	-
SOFA Score	ISTH Overt DIC	N/A	N/A	N/A	-
	JAAM DIC	1	N/A	N/A	Score assesses multi-organ dysfunction
	ISTH SIC	1	≥2	N/A	-

^1^ A reduction of 50% in platelet count within 24 h is indicative of high risk for DIC as per ISTH guidelines. ^2^ A ‘Moderate increase’ in FDP/D-dimer generally refers to a 10–25% increase from baseline levels. ^3^ A ‘Strong increase’ in FDP/D-dimer generally refers to an increase greater than 25% from baseline levels. DIC: Disseminated Intravascular Coagulation, a severe disorder causing abnormal blood clotting. SIC: Sepsis-Induced Coagulopathy, a condition where blood clotting is triggered by infection. JAAM: Methodology developed by the Japanese Association for Acute Medicine. ISTH: Methodology established by the International Society on Thrombosis and Haemostasis. PT-INR: Prothrombin Time to International Normalized Ratio, a standardized measure of blood clotting time. Measured in seconds or as a PT to International Normalized Ratio (PT-INR). SIRS: Systemic Inflammatory Response Syndrome, an indicator of systemic inflammation. SOFA: Sequential Organ Failure Assessment, an evaluation of multi-organ functionality. Platelet Count: Measured in ×10^9^ per liter (L); D-dimer Levels: Measured in micrograms per milliliter (μg/mL); Fibrinogen Levels: Measured in grams per milliliter (g/mL); “Score = 1” denotes Low-Risk Criteria, “Score = 2” denotes Moderate-Risk Criteria, and “Score = 3” denotes High-Risk Criteria. ‘N/A’ signifies that the criteria are not applicable under the particular diagnostic method.

**Table 3:** Summary of Therapies for Sepsis-Related DIC.

Therapy	Mechanism of Action	Dosage and Administration	Efficacy	Adverse Effects	References
Unfractionated Heparin (UFH)	Anticoagulant	Dosage: Based on weight, typically 80 units/kg bolus followed by 18 units/kg/hr infusion	Limited high-quality evidence for use in sepsis-related DIC. Small trials show potential benefits in early-stage sepsis patients but not necessarily in sepsis DIC patients	Bleeding risk	[19,39,40,41]
Recombinant Soluble TM (rsTM)	Alleviates DIC and reduces mortality	Dosage: Varies, typically administered intravenously	More effective than UFH in alleviating DIC and reducing mortality in infectious DIC patients	NS *	[39,40,41,42,43]
Activated Protein C (APC)	Anticoagulant and anti-inflammatory agent; degrades extracellular histones	Dosage: Varies, typically administered intravenously	No significant difference in response rates compared to UFH for DIC; reduces bleeding risk and mortality	Bleeding risk	[44,45,46,47,48,49]
High-dose Antithrombin (AT)	Reduces mortality in DIC patients without significant bleeding events	Dosage: Varies, typically administered intravenously	No reduction in mortality in sepsis patients; increases bleeding risk	Increased bleeding risk	[44,45,49,50]
Corticosteroids	Unclear mechanism; potential benefits in sepsis-induced DIC	Dosage: Varies depending on the specific corticosteroid used and patient condition	Contrasting findings, inconclusive evidence. Some studies suggest potential benefits, while others show no significant impact or potential harm	Potential adverse effects: increased risk of infection, metabolic disturbances	[32,33,34,35,36,38,51]
Thrombomodulin alfa (rTM)	Binds to thrombin, activates protein C, downregulates coagulation	Dosage: Varies, typically administered intravenously	Reduction in overall mortality rates, minimized bleeding complications	NS *	[8,52,53]
Vitamin C	Potential antioxidant, anti-inflammatory, and anticoagulant properties	Dosage: Varies, typically administered intravenously	Inconclusive evidence. Some studies show potential benefits in certain parameters, while others show no significant impact or potential harm	NS *	[54,55,56,57,58,59,60,61]
Fibrinolytic Therapy	Reduces clot formation, improves organ perfusion	Dosage: Varies depending on the specific fibrinolytic agent used	Impact on clinical outcomes inconclusive; some studies show improvements in coagulation parameters, while others show no significant effect	Bleeding risk	[62,63,64,65,66,67]
Platelet Transfusion	Controversial; potential benefits in severe thrombocytopenia or active bleeding	Dosage: Varies depending on the patient’s platelet count and clinical condition	Evidence supporting efficacy is sparse; conflicting recommendations	Potential adverse effects: bleeding complications	[68,69,70,71,72,73]
Granulocyte Colony-Stimulating Factor (G-CSF)	Stimulates production and mobilization of neutrophils	Dosage: Varies, typically administered subcutaneously or intravenously	Potential benefits in improving coagulation parameters	NS *	[74,75,76,77]
Granulocyte-Macrophage Colony-Stimulating Factor (GM-CSF)	Acts on neutrophils and monocytes/macrophages	Dosage: Varies, typically administered subcutaneously or intravenously	Impact on sepsis-induced DIC not yet clearly defined	NS	[74,75]
Interferon-gamma (IFN-γ)	Improves coagulation abnormalities, shows a trend toward decreased mortality in sepsis-induced coagulopathy patients	Dosage: Varies, typically administered intravenously	Improved coagulation abnormalities, reduced DIC duration, potential decrease in mortality	NS	[53]
Mesenchymal Stem Cells (MSCs)	Immunomodulatory effects through cytokine secretion	Dosage: Varies, typically administered intravenously	Promising results in preclinical studies, potential to improve outcomes in sepsis-induced DIC	NS *	[78,79,80,81,82,83,84,85,86,87]

* NS stands for Not Specified.

**Table 4:** Comparative Analysis of Clinical Trials and Guidelines on Coagulation Interventions in Sepsis.

Aspect	PROWESS Trial	KyberSept Trial	OPTIMIST Trial	SSCG 2021 Guidelines	Should We Target Coagulation Abnormalities?
Primary Object	Evaluate activated protein C (drotrecogin alfa) for severe sepsis	Assess high-dose antithrombin therapy in severe sepsis	Study tifacogin, a recombinant tissue factor pathway inhibitor, in sepsis	Update clinical guidelines for sepsis and septic shock	N/A
Key Findings	No long-term benefit; risk of bleeding	No benefit; potential interaction with other anticoagulants like heparin	No benefit; complexity of sepsis noted	Eliminated pharmaceutical recommendations and omitted the term “DIC”	Subject of ongoing debate
Reasons for Failure	Risk-benefit profile questioned due to bleeding risks	Possible interaction with other anticoagulants like heparin	Sepsis too complex for single-target therapy	N/A	Complexity and heterogeneity of sepsis
Scientific Implications	Raised questions about the role of anticoagulants in sepsis treatment	Highlighted the need to understand the interaction between anticoagulants	Called for a broader understanding of sepsis beyond coagulation abnormalities	Indicates shift in understanding of sepsis and coagulopathy	Raised questions about the feasibility of targeting coagulation abnormalities
Considerations for Future Research	Need for trials with better risk stratification	Exploration of combination therapies	Need for multi-targeted approaches	N/A	Requires a more nuanced approach considering the multifaceted nature of sepsis

This table summarizes and compares the primary objectives, key findings, reasons for failure, scientific implications, and considerations for future research across the PROWESS Trial, KyberSept Trial, OPTIMIST Trial, SSCG 2021 Guidelines, and the debate surrounding the targeting of coagulation abnormalities in sepsis management.

**Table 5:** Comparative Analysis of Bleeding Risks in Different Anticoagulant Studies Involving Severe Sepsis and Septic DIC Patients.

Study Name & Reference ID	Investigational Agent & Target Patient Population	Study Design & Participant Count	Classification of Bleeding Adverse Events	Incidence in Intervention Arm (%)	Incidence in Control Arm (%)	Statistical Significance	Remarks	Ref.
PROWESS	Recombinant Activated Protein C (rAPC); Patients with Severe Sepsis	Randomized Controlled Trial (RCT); N = 1690	Any Type/Major	Any: 12.5%, Major: 3.5%	Any: 12.1%, Major: 2.0%	Any: *p* = 0.84, Major: *p* = 0.06	Treatment and control groups are similar in bleeding rates	[144,150]
PROWESS-SHOCK	rAPC; Patients with Severe Sepsis and Shock	RCT; N = 1666	Any Type/Major	Any: 8.6%, Major: 1.2%	Any: 4.8%, Major: 1.0%	Any: *p* = 0.002, Major: *p* = 0.81	Significant increase in any type of bleeding; no significant difference in major bleeding.	[29,144]
KyberSept	High-dose Antithrombin; Patients with Severe Sepsis	RCT; N = 2314	Any Type/Major	Any: 22.0%, Major: 10.0%	Any: 12.8%, Major: 5.7%	RR: 1.71 (95% CI: 1.42–2.06), Major: RR: 1.75 (95% CI: 1.32–2.33)	Significantly higher bleeding risk associated with antithrombin; caution advised.	[49,144]
Iba et al.	Supplemental-dose Antithrombin; Patients with Septic DIC	Non-Randomized Phase-4 Trial; N = 729	Any Type/Major	Any: 6.52%, Major: 1.71%	N/A	N/A	Control group absent; no comparative statistical analysis possible.	[144,151]
Online Data	Recombinant Thrombomodulin (rTM); Patients with Severe Sepsis	Phase-2B Trial; N = 741	Major	Major: 6.7%	Major: 6.2%	Not Reported	Lack of significant statistical evaluation on bleeding.	[144,152]
Post-marketing Survey	Recombinant Thrombomodulin (rTM); Patients with Septic DIC	Phase-4 Trial; N = 2516	Any Type	Any: 5.4%	N/A	N/A	Survey study; No control group for comparison. Possible.	[144,152]

Randomized Controlled Trial (RCT) is a robust study design featuring both an intervention and a control arm for unbiased comparison. Non-randomized and phase trials lack a control arm, limiting their comparative statistical validity. Statistical significance (*p*-value or Relative Risk with Confidence Interval) is used to assess whether the observed differences are likely to be due to the intervention rather than random variability. Abbreviations: rAPC: Recombinant Activated Protein C; rTM: Recombinant Thrombomodulin; DIC: Disseminated Intravascular Coagulation; RCT: Randomized Controlled Trial; RR: Relative Risk; CI: Confidence Interval.

## Data Availability

Not applicable.
